# Tuberculosis screening among pregnant women attending a tertiary care hospital in Puducherry, South India: is it worth the effort?

**DOI:** 10.1080/16549716.2018.1564488

**Published:** 2019-02-26

**Authors:** Mathavaswami Vijayageetha, Ajay MV Kumar, Jayalakshmy Ramakrishnan, Sonali Sarkar, Dasari Papa, Kedar Mehta, Noyal M Joseph, Manju Rajaram, Sathish Rajaa, Palanivel Chinnakali

**Affiliations:** a Department of Preventive and Social Medicine, Jawaharlal Institute of Postgraduate Medical Education and Research (JIPMER), Puducherry, India; b Center for Operational Research, International Union Against Tuberculosis and Lung Disease, Paris, France; c South-East Asia Office, International Union Against Tuberculosis and Lung Disease, New Delhi, India; d Department of Obstetrics and Gynaecology, Jawaharlal Institute of Postgraduate Medical Education and Research (JIPMER), Puducherry, India; e Department of Community Medicine, GMERS Medical College, Vadodara, India; f Department of Microbiology, Jawaharlal Institute of Postgraduate Medical Education and Research (JIPMER), Puducherry, India; g Department of Pulmonary Medicine, Jawaharlal Institute of Postgraduate Medical Education and Research (JIPMER), Puducherry, India

**Keywords:** Antenatal women, active case finding, intensified case finding, SORT IT, operational research

## Abstract

**Background**: The national tuberculosis (TB) programme in India recommends screening all pregnant women for TB, but this is rarely implemented. We carried out systematic TB screening among women attending the antenatal clinic of a tertiary care hospital in Puducherry, South India, during February to April 2018.

**Objective**: To assess the number screened and number (proportion) with presumptive and active TB, and understand potential implementation from the healthcare providers’ perspective.

**Methods**: We conducted a mixed-methods study. The quantitative phase was a cross-sectional study including 4203 pregnant women. Data were captured using a structured proforma. Any of the following symptoms constituted ‘presumptive TB’: any cough, haemoptysis, fever, weight loss, night sweats, neck swellings, joint pains, neck stiffness and disorientation. Those screening positive were referred for investigations and evaluation by a chest physician. The qualitative phase involved seven one-to-one interviews with healthcare providers. Manual thematic analysis was performed to generate themes.

**Results**: Among 4203 women (two HIV-positive) screened, 77 (1.8%) had presumptive TB. Cough was the predominant symptom (*n* = 75). Only 12 women could produce a sputum sample, of whom one (0.02%) was diagnosed with active TB by the Xpert MTB/RIF assay. Challenges cited by healthcare providers were lack of awareness among clients and providers, high case load, lack of dedicated staff, perception that TB screening is a low-yield, low-priority activity and losses in the referral process. Suggested solutions were providing dedicated staff and space for screening, educating women to self-report using posters and videos, and creating a one-stop care provision.

**Conclusions**: The TB yield among pregnant women was very low, calling into question the value of systematic screening in a low-HIV setting. However, the findings may not be generalizable. Evidence is urgently needed from primary and secondary care facilities. The challenges and solutions identified may help in optimizing the screening process.

## Background

Tuberculosis (TB) is one of the most important infectious causes of maternal mortality globally and accounts for 16% of all maternal deaths [1]. India contributes to nearly 21% of the global burden of TB among pregnant women and the estimated prevalence of TB stands at 2.3 per 1000 pregnant women, which translates to about 44,500 patients annually [2]. TB among pregnant women is associated with unfavourable maternal and foetal outcomes, evidenced by a six-fold increase in perinatal deaths and a two-fold increase in preterm births and low birth weight [3–5]. Among pregnant women living with human immunodeficiency virus (HIV), TB increases the risk of maternal and infant mortality by an additional 300% [6]. Therefore, the World Health Organization (WHO) recommends systematic screening for active TB in pregnant women within routine healthcare services such as antenatal care programmes, where the TB prevalence in the general population is 100/100,000 population or higher [7].

**Figure 1.**
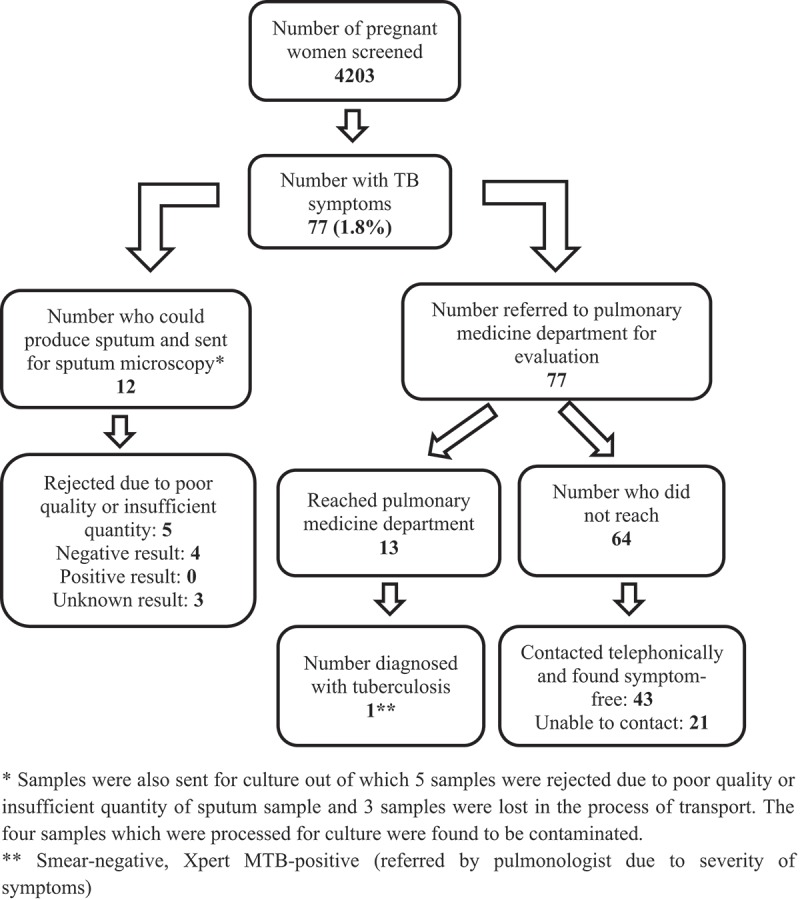
Process and results of tuberculosis (TB) screening among pregnant women attending a tertiary care hospital in Puducherry, South India, between February and April 2018.

**Figure 2.**
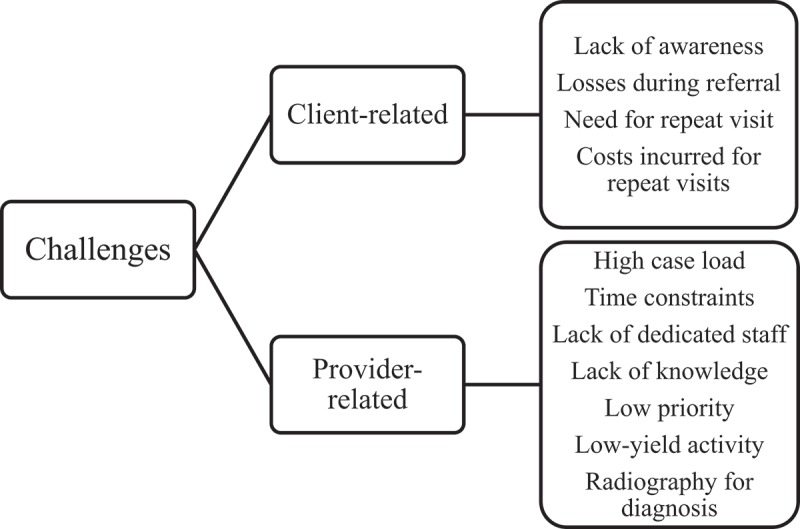
Challenges (as perceived by healthcare providers) in implementing systematic screening for tuberculosis among pregnant women attending a tertiary care hospital in Puducherry, South India.

The Revised National Tuberculosis Control Programme (RNTCP) in India recommends that all pregnant women undergo symptom screening at every antenatal visit, since this provides an opportunity to screen for pulmonary TB and facilitate early diagnosis and treatment [8]. Anecdotal evidence indicates that this recommendation is rarely implemented and there is no system of recording, reporting or monitoring. The reasons for poor implementation are unknown.

Much of the evidence supporting the benefits of systematic screening for TB through integrated services has been reported from settings with a high prevalence of HIV and high TB in Kenya and South Africa [9,10]. There have been only two studies on this issue from India [11,12]. One study included only HIV-positive pregnant women and found a TB prevalence of 1.4%, clearly indicating that TB screening among people with HIV is worthwhile [11]. In another study, which included both HIV-positive and HIV-negative pregnant women, 209 women were screened and nine women were reported to have had TB infection or disease [12]. It is not clear how many of these nine women had TB infection only and how many had active TB disease. Four of these nine women were HIV positive, once again showing the value of TB screening among HIV-positive pregnant women.

Even in high-HIV settings in Africa, the yield of TB is very low among HIV-negative pregnant women [10,13,14]. Therefore, it is not clear whether TB screening should be targeted among HIV-positive pregnant women only or among all pregnant women.

Studies from Africa indicate that many challenges exist while integrating screening for TB in pregnant women, such as non-specific symptoms, potential effects of radiation, which may limit the use of radiography, acceptability, difficulty in producing a second sputum sample, lack of transportation and distance to travel, and providers’ fear of contracting TB from collected sputum samples [4,15,16]. However, there is limited evidence on implementation challenges from programmatic settings in India.

Therefore, we decided to implement TB screening among pregnant women attending the antenatal care clinic in a tertiary care hospital of South India for 3 months (February to April 2018) to determine of the yield of TB and understand the implementation challenges. In this paper, we report the numbers screened, and number (proportion) with presumptive and active TB, and explore, from the healthcare providers’ perspective, the feasibility and potential challenges of implementing the TB screening, and possible solutions.

## Materials and methods

### Study design

This was a convergent mixed-methods study design with a cross-sectional quantitative component and a descriptive qualitative component [17].

### Setting and study site

The study was conducted at the antenatal clinic located in the Department of Obstetrics and Gynaecology (OG) of the Jawaharlal Institute of Postgraduate Medical Education and Research (JIPMER), a tertiary care facility located in the Puducherry district of South India. JIPMER provides state-of-the-art, multi-speciality healthcare services, largely to the south Indian population, and has a bed capacity of 1840. The daily average outpatient attendance stands at 7827. There is a designated microscopy centre, located in the Pulmonary Medicine Department (PMD), where sputum microscopy is performed. It also houses a GeneXpert machine, which is mainly used for diagnosis of paediatric TB, HIV-associated TB, extrapulmonary TB and multidrug-resistant TB. Patients from all departments are referred and evaluated as per RNTCP guidelines.

On average, about 300 pregnant women access care at the antenatal clinic at JIPMER every day, of whom about 40 women are newly registered. Most antenatal women visiting the clinic are referred from other peripheral health facilities for specialized care and include a large proportion (around 60%) of high-risk pregnancies. Every antenatal woman is provided with a package of services, which include registration with a unique hospital identification number (UID), clinical examination, consultant advice, screening for high-risk pregnancy, counselling services, investigations to check for haemoglobin, blood group, HIV, oral glucose tolerance test, hepatitis B surface antigen and venereal disease research laboratory tests, iron and folic acid supplementation, follow-up, birth preparedness and a plan for delivery.

All services are provided free of charge for pregnant women, irrespective of their economic status.

### Process of screening for TB

The principal investigator (PI) trained 10 staff (medical social workers and nurses, who had been hired for another TB-related research project) to conduct the screening. Screening was carried out by one to three staff, depending on their availability, on any given day. Every pregnant woman was screened only once during the study period. After screening, the woman’s case file was stamped as ‘screened’. During subsequent visits, the stamp helped in identifying the pregnant women who had already been screened and prevented repeat screening.

All pregnant women were systematically asked whether they had TB symptoms (cough, blood in sputum, fever, loss of weight or inability to gain weight, night sweats, swelling of lymph nodes in the neck, joint pain and swelling, neck stiffness, disorientation), along with their duration, using a checklist. Participants who screened positive for any of the symptoms were considered to have ‘presumptive TB’.

One or two ‘spot sputum samples’ were collected from the presumptive cases, 1 hour apart and in a sterile container, and transported within 4 hours to the Department of Microbiology for sputum microscopy (Ziehl–Neelsen technique) and culture (in Löwenstein–Jensen medium). All presumptive cases of pulmonary TB and extrapulmonary TB were referred to the PMD for further evaluation. The Xpert MTB/RIF assay was used in diagnosis if there was a high index of suspicion, based on the discretion of the chest physician.

Women who were smear positive for acid-fast bacilli, or culture positive for *Mycobacterium tuberculosis* or positive on the Xpert MTB/RIF assay were considered as ‘bacteriologically confirmed TB’. Those found to have negative sputum smears were evaluated for clinical TB. Those with presumptive extrapulmonary symptoms were evaluated, based on the affected site. The diagnosed TB patient was then referred and linked to the nearest health facility for initiation of treatment as per the RNTCP guidelines.

### Study population

#### Quantitative

All pregnant women, both newly and previously registered, who attended the antenatal clinic and underwent screening for TB between February and April 2018 were included.

The sample size was calculated using OpenEpi software version 3.01. Assuming an estimated TB prevalence of 0.23%, a precision of 0.12%, a 95% confidence level and a finite population correction factor for an assumed finite population of 10,000, a sample size of 3798 was obtained [2].

#### Qualitative

Healthcare providers involved in the care of pregnant women were interviewed. We used purposive sampling to ensure representation of various cadres of staff involved. These included an administrator, obstetricians, chest physicians and nursing officials.

### Data variables, sources of data and data collection

#### Quantitative data collection

A structured questionnaire was used to collect data. The variables included baseline characteristics (UID, age in years), obstetric characteristics (gestational age in weeks), clinical characteristics [haemoglobin in g/dl, HIV status, gestational diabetes mellitus (GDM), pregnancy-induced hypertension (PIH), syphilis and any other high-risk conditions], previous history of TB, current history of contact with a known case of TB, and results of symptom screening for pulmonary and extrapulmonary TB [8].

Information on the baseline and epidemiological characteristics was collected directly from the study participants and clinical characteristics were extracted from the hospital case sheets. The results of the investigations were extracted from the laboratory registers maintained at the Department of Microbiology using UIDs and communicated to the pregnant women by telephone. The information on clinically diagnosed pulmonary TB and extrapulmonary TB was extracted by reviewing the ‘Referral for treatment’ register maintained at the PMD and the Hospital Information System records of the hospital. All the proforma were rechecked by the PI for completeness and assessed for consistency of the collected data.

To ensure that we had not missed any TB patients among the pregnant women who did not reach the PMD for evaluation, we telephoned them at the end of study to assess whether they had received care for presumptive TB symptoms elsewhere, and the outcome of that care (whether symptom free or not).

#### Qualitative data collection

Seven key informant interviews were conducted and saturation was used to guide the sample size [18]. The participants were one senior administrator, two senior nursing officers, one chest physician, two resident doctors from OG and one resident doctor from PMD. The mean duration of interviews was 31 minutes (range 23–42 minutes). The interview was conducted by the PI (a female medical doctor, trained in qualitative research). She had good knowledge of the setting, but was not involved in the provision of care for pregnant women. Interviews were conducted at a date, time and place convenient to the participants after establishing rapport. The PI held face-to-face interviews using an interview guide with broad open-ended questions and probes. Audio recording was done and verbatim notes were taken during the interview. After the interview was over, the key messages were summarized to the participants to ensure participant validation. In addition to interviews, observations made by the staff involved in the screening process were captured as field notes.

### Analysis and statistics

#### Quantitative

Quantitative data were double-entered, validated and analysed using EpiData (version 3.1 for entry and version 2.2.2.183 for analysis; EpiData Association, Odense, Denmark). Key analytical outputs were the number and proportion of presumptive TB patients, and the number and proportion of active TB disease. Yield was calculated by dividing the number of TB patients by the total number of women screened. Multivariable analysis could not be performed because of insufficient cases of active TB.

#### Qualitative

Transcripts were prepared within 3 days of the interview by VG and SR. Manual thematic analysis was used to analyse data. Initial coding and theme generation were carried out independently by two investigators (VG and KM). Similar basic themes were grouped as organizing themes and then into a global theme, utilizing a thematic network analysis method as described by Attride-Stirling, and represented as a non-hierarchical figure [19]. The analysis was reviewed by a third investigator (AMVK) to reduce bias and subjectivity in interpretation of the findings. Any differences were resolved by discussion. The findings were reported using ‘Consolidated Criteria for Reporting Qualitative Research’ [20].

## Results

### Quantitative

#### Baseline characteristics

In total, 4203 pregnant women were screened. Only two women (0.05%) were HIV infected. About 90% of women belonged to the 20–34-year age group, and nearly 60% were in the third trimester of pregnancy. (Table 1) Two-thirds of women had anaemia. Twenty-seven women (0.6%) had a past history of TB and 16 (0.4%) were currently living with a case of TB.

**Table 1.** Baseline characteristics of antenatal women screened for tuberculosis in a tertiary healthcare facility, Puducherry, India, between February and April 2018 (*n* = 4203).

Characteristics	Category	Frequency	Proportion
Age (years) (*n* = 3831)*			
	15–19	245	6.4
	20–34	3477	90.8
	35–45	109	2.8
	Mean (SD)	24.8 (4.1)	
Gestational age (weeks) (*n* = 4094)*			
	1st trimester (≤12 weeks)	190	4.6
	2nd trimester (13–28 weeks)	1530	37.4
	3rd trimester (>28 weeks)	2374	58.0
	Mean (SD)	28.8 (7.9)	
Risk status			
	High risk	2847	67.7
	Normal	1356	32.3
High-risk condition			
	Anaemia	2660	63.3
	HIV	2	0.1
	GDM	73	1.7
	HBsAg	34	0.8
	PIH	30	0.7
	Cardiac disease	15	0.4
	Recurrent pregnancy loss	38	0.9

*The numbers are less than the total sample owing to missing values.

HIV, human immunodeficiency virus; GDM, gestational diabetes mellitus; HBsAg, hepatitis B surface antigen; PIH, pregnancy-induced hypertension; SD, standard deviation.

### Presumptive TB

Of 4203 women screened, 77 (1.8%) had at least one of the symptoms qualifying for presumptive TB (Table 2). Cough was the predominant symptom, observed in 75 women (1.8%), of whom 19 (0.4%) had had a cough for 2 or more weeks. Other symptoms were rare.

**Table 2.** Presumptive tuberculosis (TB) symptoms presented by antenatal women screened in a tertiary healthcare facility, Puducherry, India, between February and April 2018.

Symptom	Frequency	Proportion
Total	4203	100
Presumptive TB	77	1.8
Cough	75	1.8
<1 week	24	0.6
1–2 weeks	32	0.8
≥2 weeks	19	0.4
Haemoptysis	1	0.02
Fever	2	0.05
Weight loss	1	0.02
Inability to gain weight	1	0.02
Night sweats	0	0
Neck swellings	1	0.02
Joint pain or swelling	1	0.02
Neck stiffness	1	0.02
Disorientation	0	0

### *TB investigation and diagnosis* (Figure 1)

Among 77 women with presumptive TB, 12 (16%) were able to produce a sputum sample. Of these, five samples were rejected by the laboratory owing to poor quality or insufficient quantity or both, and another three samples were lost in transit. None of those examined was smear positive. All women with presumptive TB were also referred to the chest physician for further evaluation, of whom 13 (16.9%) turned up. Among those who attended the referral, only one case of active pulmonary TB was detected using Xpert MTB/RIF. Thus, the yield of screening was 0.02%. The remaining 64 women (83.1%) who did not attend were contacted by telephone. Of 43 women who could be contacted by telephone, all had become symptom free after receiving treatment from private providers or traditional medicine practitioners.

### Qualitative

#### Feasibility

All the healthcare providers interviewed unanimously acknowledged the importance of screening for TB in pregnant women. Most felt that it helps in early diagnosis and treatment and prevents adverse maternal and perinatal outcomes.

However, with regard to the feasibility of implementation, the providers had varied views. Some providers felt that screening was feasible because, in their view, it did not take much time to ask a few questions about TB symptoms. Some providers strongly felt that screening was not feasible because they thought that this would add to the already high workload of caring for hundreds of pregnant women every day:

I don’t think it’s possible … sitting and asking for each patient regarding the symptoms when we have an OPD [outpatient department] with 500 to 600 patients, in five hours you have to finish it. So, on an average 100 patients you have to see an hour. It’s not possible to sit and screen all these patients. (OG resident)

Some providers felt that it was conditionally feasible, if dedicated staff and space were provided to conduct the screening.

### Potential challenges in implementation

Challenges were grouped into two broad categories: client-related and provider-related. (Figure 2)

#### Client-related challenges

Healthcare providers perceived a lack of awareness among pregnant women about the need for TB screening as a key challenge. Other challenges noted were the need to visit different departments for clinical evaluation, the necessity for repeat visits for undergoing tests for TB and the costs incurred.

#### Provider-related challenges

The most common challenge cited was the high case load and the increased burden it places on existing staff. The other related challenge was a lack of dedicated staff to conduct the screening. Some cited time constraints in asking for all the symptoms at every visit:

One or two patients, fine. The same thing for everybody, I don’t think I will do justice for it, mainly because of the patient load. (OG resident)

Some noted that not all healthcare providers are aware of the protocol for TB screening among pregnant women (how to screen, when to screen, what tests are to be done for diagnosis, where to refer for treatment, how the patient should be managed at delivery, and so on).

Some healthcare providers felt that TB ranks low in priority among pregnant women, given the low incidence of TB in this group, compared to more common conditions such as anaemia, PIH and GDM. One resident mentioned that TB screening is ‘unrewarding for the effort’ put in.

Another challenge was related to the losses that happen when women are referred from one department to another for investigations, and the lack of a mechanism to follow up such women.

Many providers mentioned the challenge of using chest radiography for screening given the possibility of radiation hazards to the foetus, particularly in the first trimester. However, most were optimistic that radiography may be used in the second and third trimesters, if required, using an abdominal shield.

### Suggested solutions

Many valuable solutions were suggested by healthcare providers to address the challenges.

#### Increase awareness

One suggestion was to increase awareness among the pregnant women by distributing pamphlets, displaying posters and playing videos in the waiting areas. Healthcare providers also suggested that a self-administered symptom checklist (in local language) could be provided to each pregnant woman, which could be filled during the waiting time and shared with the doctor at the time of consultation.

One of the providers also felt the need to increase awareness among medical personnel and paramedics:

We only think about anaemia or heart diseases. Respiratory symptoms are never given importance. So awareness has to be created among the doctors and paramedics to screen for these symptoms. (Chest physician)

#### Appoint dedicated staff

Many providers suggested the need to appoint dedicated staff for screening, as has been done by the National AIDS Control Programme for HIV testing and counselling. They also suggested involving existing staff (nurses or other paramedics) in TB screening at the point of registration.

#### One-stop care

To avoid losses in the referral process, some providers suggested that all care should be provided at a single point, by either posting a chest physician on a rotational basis in the antenatal clinic or training obstetricians for screening and further management. It was suggested that sputum samples may be collected at the antenatal clinic and transported to the laboratory for prioritized examination.

### Field observations

Several observations were made by the staff involved in screening. They noted that the antenatal clinics were overcrowded and it was not possible to cover all the pregnant women because of the overwhelming numbers and lack of a dedicated space for interacting with them. The staff felt that they were able to screen only about 40–50% of the pregnant women during the initial months. This improved gradually to about 80–85% in the final month of the study, as many of the women had already been screened.

Some pregnant women did not cooperate with the staff involved in screening, as they were trying to obtain a consultation with the doctor as a priority and feared waiting in the queue for long hours. It was noted that some of them bypassed the registration process and consulted the doctors directly. Many women with presumptive TB were unable to produce sputum and even among those who were able to, the quantity and quality of sputum were poor. Lack of a dedicated space for sputum collection, inadequate time for individual counselling, and the discomfort and exertion of women while trying to bring up sputum were also observed.

## Discussion

This study from India adds to the global evidence base reporting on the challenges in implementing TB screening among all pregnant women (both HIV positive and negative). The study had several interesting findings.

First, the yield of TB was abysmally low (0.02%) and a whopping 4203 women had to be screened to find one case of TB. This is one of the lowest yields of TB reported among pregnant women, barring a study from Ethiopia which reported a yield of 0.01% [21]. In contrast, the yield of TB varied from 0.1% to 1.6% in previous studies from Kenya, South Africa, Tanzania, Zambia and India [10,13,21–23]. The variation in yield might be explained by the differences in study population (whether only HIV-positive or all pregnant women were included), HIV prevalence, definitions of TB used and the background TB prevalence.

The low yield of TB may be one of the reasons why TB screening among pregnant women remains neglected. This was noted by one of the doctors interviewed, who said that compared to other common conditions during pregnancy, such as anaemia, PIH and GDM, TB ranks low in priority and perhaps is ‘unrewarding for the effort’.

This calls into question the value of systematic screening in a low-HIV setting. However, based on this, can we conclude that TB screening should not be conducted routinely among pregnant women (especially among HIV-negative women)? We feel that the evidence is insufficient for us to arrive at a definitive conclusion, for several reasons.

First, our study was conducted in one tertiary care hospital. So, caution is required before generalizing the findings to other settings with a much lower case load. Future research should focus on conducting investigations in primary and secondary care facilities.

Secondly, we faced many challenges in implementation which meant that not all presumptive TB patients were optimally evaluated for bacteriological confirmation. The majority of the women had non-productive cough and were not able to produce sputum. In women who managed to expectorate after exertion, the quality and quantity were poor. Although we referred all presumptive TB cases to the PMD, only one in six attended. We were worried about the possibility of missing TB in the group that did not attend and were not evaluated by a chest physician. Therefore, we telephoned all the women at the end of the study to find out about their health, and all those contacted informed us that they were symptom free, providing reassurance that we were not missing TB.

Thirdly, TB may be rare among pregnant women, but when it occurs, it leads to many complications including the death of the mother and the newborn. A recent study from the USA (a low TB prevalence setting) shows that TB in pregnant women increased the risk of complications by 80% and increased the risk of death by 37-fold [24]. Thus, the cost of missing TB among pregnant women is too high to take the stance that routine screening is not required.

The inability of pregnant women to produce a sputum sample may be related to the definition of presumptive TB used in our study, where cough of any duration was considered. While cough of any duration is a suitable criterion for people living with HIV, it may not be suitable in low-HIV settings, where more than 99% of the pregnant women are HIV negative. We suggest a higher threshold (perhaps productive cough of 2 weeks or more) for defining presumptive TB. This would also reduce the number of pregnant women classified as ‘presumptive TB’ by about 75% and save the effort and resources required for further evaluation.

Key informants interviewed had mixed views about the feasibility of implementing the TB screening, with some strongly agreeing and some strongly disagreeing. Our view is that TB screening is feasible, provided the following conditions are met.

First, dedicated staff should be provided for screening. Although some providers suggested the possibility of training existing nursing or paramedical staff, we feel that it would be challenging to cover all pregnant women, given the high case load in a tertiary care facility. In the study, even with dedicated staff, only an estimated 40–80% of the antenatal women were covered. One limitation was that we could not accurately calculate the screening coverage because of the lack of accurate information about the denominator. The medical records department collects information on the number of visits, but not on the number of women (after accounting for repeat visits). It was also noted during data collection that several pregnant women were directly consulting the doctor, bypassing the registration process, which adds to the challenge.

Secondly, dedicated space should be provided for screening. Lack of space in the overcrowded antenatal clinic was a major challenge encountered in this study.

Thirdly, aiming to screen all pregnant women at least once during pregnancy instead of at every visit makes TB screening more feasible. In our view, the best time for screening would be at the time of enrolment into antenatal care, when the pregnant women may be educated and counselled to self-report to the doctor if they develop a productive cough that does not resolve within 2 weeks. These messages need to be reinforced to the pregnant women by displaying posters and playing videos in the waiting area of the clinic.

Fourthly, increasing the threshold for screening, as discussed above, reduces the number of patients requiring investigation, thus making it more feasible to implement.

Finally, the only TB case found in our study was diagnosed because of the use of the Xpert MTB/RIF assay, which was not part of the study protocol, but was used based on the discretion of the treating chest physician. The national guideline on ‘Programmatic management of drug resistant TB’ in India now recommends the use of Xpert MTB/RIF among pregnant women with presumptive TB, given its high sensitivity, rapid turnaround and requirement for only one sputum sample. This obviates the need for repeat visits for the pregnant women, a challenge reported in our study, and saves time and costs.

One of the strengths of our study was the use of a mixed-methods design. The quantitative and qualitative methods used complemented each other and helped in providing insights into the challenges and possible solutions. However, we did not study the perspectives of pregnant women and programme managers. This should be a topic for future research. Other strengths were the large sample size, which may have minimized the random errors, and double-data entry and validation to minimize data-entry errors.

## Conclusion

The yield of TB was very low among pregnant women attending the antenatal care clinic in a tertiary care hospital in South India. This calls into question the value of systematic screening in a low-HIV setting. However, the findings may not be generalizable and evidence is urgently needed from primary and secondary care facilities. The challenges and solutions identified in this study may help in optimizing the screening process.

## Paper context

Tuberculosis in pregnancy has adverse perinatal outcomes. The WHO and RNTCP recommend screening for TB during antenatal visits. This is not widely implemented in India and the yield of TB remains unknown. Therefore, we sought to find the number of pregnant women with active TB by screening during routine visits. The findings could aid policy makers in streamlining implementation of TB screening among pregnant women. The potential challenges identified and solutions suggested could optimize screening.
